# Genetic Alterations within the *DENND1A* Gene in Patients with Polycystic Ovary Syndrome (PCOS)

**DOI:** 10.1371/journal.pone.0077186

**Published:** 2013-09-27

**Authors:** Mette B. Eriksen, Michael F. B. Nielsen, Klaus Brusgaard, Qihua Tan, Marianne S. Andersen, Dorte Glintborg, Michael Gaster

**Affiliations:** 1 Department of Endocrinology, Odense University Hospital, Odense, Denmark; 2 Department of Clinical Pathology, Odense University Hospital, Odense, Denmark; 3 Department of Clinical Research, University of Southern Denmark, Odense, Denmark; 4 Department of Clinical Genetics, Odense University Hospital, Odense, Denmark; 5 Department of Epidemiology and Biostatistics, Institute of Public Health, University of Southern Denmark, Odense, Denmark; University Hospital S. Maria della Misericordia, Udine, Italy

## Abstract

Polycystic ovary syndrome (PCOS), the most common endocrine disease among premenopausal women, is caused by both genes and environment. We and others previously reported association between single nucleotide polymorphisms (SNPs) in the *DENND1A* gene and PCOS. We therefore sequenced the *DENND1A* gene in white patients with PCOS to identify possible alterations that may be implicated in the PCOS pathogenesis. Patients were referred with PCOS and/or hirsutism between 1998 and 2011 (n = 261). PCOS was diagnosed according to the Rotterdam criteria (n = 165). Sequence analysis was performed in 10 patients with PCOS. Additional patients (n = 251) and healthy female controls (n = 248) were included for SNP genotyping. Patients underwent clinical examination including Ferriman-Gallwey score (FG-score), biochemical analyses and transvaginal ultrasound. Mutation analysis was carried out by bidirectional sequencing. SNP genotyping was tested by allelic discrimination in real-time PCR in the additional patients and controls. Sequencing of the *DENND1A* gene identified eight SNPs; seven were not known to be associated with any diseases. One missense SNP was detected (rs189947178, A/C), potentially altering the structural conformation of the DENND1A protein. SNP genotyping of rs189947178 showed significantly more carriers among patients with PCOS and moderate hirsutism compared to controls. However, due to small sample size and lack of multiple regression analysis supporting an association between rs189947178 and FG-score or PCOS diagnosis, this could be a false positive finding. In conclusion, sequence analysis of the *DENND1A* gene of patients with PCOS did not identify alterations that alone could be responsible for the PCOS pathogenesis, but a missense SNP (rs189947178) was identified in one patient and significantly more carriers of rs189947178 were found among patients with PCOS and moderate hirsutism vs. controls. Additional studies with independent cohort are needed to confirm this due to the small sample size of this study.

## Introduction

Polycystic ovary syndrome (PCOS) is the most common endocrine disorder in premenopausal women and affects up to 10% [1]. Based on the Rotterdam criteria, the PCOS diagnosis includes hyperandrogenemia (clinical/biochemical), chronic anovulation and/or polycystic ovaries when other causes have been excluded [2]. PCOS is associated with insulin resistance, resulting in a 5–8 times increased risk of type 2 diabetes (T2D) [3]. PCOS is often accompanied by hirsutism and more than 80% of hirsute patients are diagnosed with PCOS [4].

The pathogenesis of PCOS is thought to be caused by a combination of genetic and environmental factors [4,5]. Insulin resistance has been studied as a potential site of origin for the pathogenesis of PCOS [6,7] and the overlap between PCOS and T2D has led to candidate-gene based approaches focusing on T2D. A recent study found no significant associations between T2D susceptibility loci and PCOS [8]. In addition, no association was found between PCOS and single nucleotide polymorphisms (SNPs) representing susceptibility loci for T2D, metabolic or cardiovascular traits [9]. Association between SNPs in the T2D-related genes *FTO* and *MC4R* and obesity was found in patients with PCOS, however the SNPs were not associated with PCOS itself [10]. The first genome wide association study (GWAS) with Chinese patients with PCOS found association between SNPs in three loci (2p16.3: rs13405728, 2p21: rs13429458, rs12478601, rs12468394, 9q33.3: rs10818854, rs2479106, rs10986105) and PCOS [11]. The three loci were located near the gene for a G protein–coupled receptor for luteinizing hormone and human chorionic gonadotropin (*LHCGR*) and within introns of the thyroid adenoma associated gene (*THADA*) and the DENN domain containing 1a (*DENND1A*) gene [11]. A second recent GWAS also conducted with Chinese patients with PCOS confirmed the three loci from the first GWAS, and additionally identified eight new loci associated with PCOS susceptibility [12].

Despite the differences in the genetic background between Chinese and white European patients with PCOS or patients of European ancestry, the association between PCOS and SNPs in the *DENND1A* gene has been replicated in several studies [13–16] and suggests a potentially important role for *DENND1A* in the PCOS pathogenesis. The linkage between underlying molecular defects or alterations in *DENND1A* and the PCOS pathogenesis is so far undetermined.

*DENND1A*, or *connecdenn1*, encodes a protein containing a domain differentially expressed in normal and neoplastic cells (DENN), localized in the N-terminus of the protein. Several proteins harbour this domain, which is conserved between species and throughout evolution [17]. The DENND1A protein was originally described in relation to synaptic clathrin-coated vesicles in neurons. The protein is present in high levels in brain and testis [18]. In addition to the N-terminal localized DENN domain, DENND1A contains binding motifs for clathrin, the clathrin adaptor protein-2 (AP-2) and Src homology 3 (SH3) domains [18]. The conserved DENN domain of DENND1A acts as a nucleotide exchange factor for the GTPase Rab35 [19]. Rab35 is found in the plasma membrane and endocytic compartments and functions by regulating endosomal recycling. Rab35 plays a major part in cytokinesis [20].

The aim of this study was to characterize the protein-coding sequence of the *DENND1A* gene in PCOS patients and to detect any genetic alterations possibly contributing to the PCOS pathogenesis. To our knowledge, the complete coding sequence of the *DENND1A* gene of patients with PCOS has not previously been described, despite the association between variation in *DENND1A* and PCOS, reported by us and others. The sequence analysis of *DENND1A* did not identify alterations that alone could be the PCOS pathogenesis; however a missense SNP was detected in one patient. Further analysis in additional patients and controls showed significantly more carriers of this SNP among patients with PCOS and moderate hirsutism compared to controls. The rs189947178 missense SNP may represent a part of a rare PCOS genotype or it may be a marker for PCOS related genetic alterations located apart from *DENND1A.*

## Materials and Methods

### Ethics statement

The study was approved by the local ethics committee (The Scientific Ethical Committee for Vejle and Funen Counties now referred to as The Scientific Ethical Committee of the Region of Southern Denmark) and all subjects gave written informed consent.

### Patients and controls

In a previous study, the rs2479106 G allele was associated with decreased PCOS susceptibility in a cohort of white patients with PCOS [16]. The study cohort consisted of 261 white, female patients referred to Department of Endocrinology, Odense University Hospital, Denmark, with PCOS or hirsutism. The included patients were characterized by clinical hyperandrogenism only (n = 96) and/or fulfilled the Rotterdam criteria for PCOS (n = 165) [21].

Two hundred and forty eight healthy, white, pre-menopausal women were recruited as controls from the local area of Funen during the same period of time, and all with regular menstruations (cycle length of 26–34 days) and no complaints of hirsutism were included as controls [16,22]. Two hundred and nine controls were blood donors (initially, 345 women gave informed consent and completed a questionnaire concerning menstrual cycles and hirsutism. 136 female blood donors were excluded: 86 due to hysterectomy/menopause, 42 due to hirsutism and 8 blood donors due to irregular menstruations). Thirty nine controls were included from a study aimed to establish a reference interval for 17OHP responses during the ACTH test [22]. These women underwent clinical examination and were characterized by total testosterone < 1.8 nmol/l and maximum total Ferriman-Gallwey score ≤ 1.

The patient- and control cohorts have been thoroughly described in previous studies [16,22]. Patients and controls paused oral contraceptives for at least three months before evaluation and did not use medicine known to affect hormonal or metabolic parameters.

We defined a Ferriman-Gallwey score ≥ 16 (n = 47) as the cut-off for moderate hirsutism, according to the 95th percentile in unselected populations of premenopausal white women [23–29]. PCOS (n = 165) was defined according to the Rotterdam criteria [2].

From the patient cohort, we selected ten patients with PCOS for direct sequencing of the *DENND1A* gene (table 1). The ten sequenced patients were randomly selected from 29 patients that fulfilled the following criteria: the patients fulfilled all three of the Rotterdam criteria [2], had free testosterone > 0.034 nmol/l and a Ferriman-Gallwey score ≥ 7 [30].

**Table 1:** Clinical and biochemical characteristics of patients with PCOS and controls.

	**Controls (n = 39)**	**Patients (n = 261)**	**Unsequenced PCOS (n = 155)**	**Sequenced PCOS (n = 10)**
**Age (years)**	25 (23-27)	31 (25-36)^**^	29 (24-34)	30 (25-32)
**Weight (kg)**	65.0 (60.0-73.0)	72.8 (64.1-86.8)^**^	72.8 (64.1-87.6)	88.8 (76.8-101.0)†
**BMI (kg/m^2^)**	23.3 (21.6-24.8)	26.0 (22.6-30.6)^**^	26.1 (22.7-30.6)	30.5 (27.0-38.4)†
**WHR**	0.73 (0.70-0.77)	0.82 (0.76-0.86)^**^	0.82 (0.77-0.87)	0.86 (0.79-0.93)
**Ferriman-Gallwey score**	0 (0-1)	10 (6-14)^**^	10 (6-13)	15 (10-21)†
**Free testosterone (nmol/l)**	0.019 (0.013-0.024)	0.033 (0.021-0.048)^**^	0.034 (0.023-0.053)	0.049 (0.039-0.066)†
**SHBG (nmol/l)**	65 (51-84)	54 (37-74)^*^	51 (35-72)	33 (18-38)†
**Fasting glucose (mmol/l)**	4.9 (4.6-5.2)	4.6 (4.3-5.0)^*^	4.8 (4.3-5.2)	4.7 (4.1-4.8)
**Fasting insulin (pmol/l)**	31 (20-40)	52 (36-82)^**^	52 (38-83)	82 (57-98)
**Triglyceride (mmol/l)**	0.8 (0.6-1.0)	1.1 (0.8-1.5)^**^	0.9 (0.7-1.4)	1.4 (1.3-2.3)†
**HDL (mmol/l)**	1.5 (1.3-1.8)	1.3 (1.1-1.6)^*^	1.3 (1.2-1.6)	1.4 (1.1-1.6)
**LDL (mmol/l)**	2.3 (1.9-2.6)	2.9 (2.4-3.4)^**^	2.8 (2.3-3.3)	3.3 (2.5-4.3)

Data are presented as median (25-75% quartiles). P-values were calculated by Mann-Whitney test. These results have partly been published before [16,21,22].

* P < 0.05 - patients vs. controls.

** P < 0.001 – patients vs. controls.

† P< 0.05 - sequenced PCOS vs. PCOS.

Recruiting family members was avoided.

### Clinical and biochemical analyses

Routine evaluation included clinical evaluation, blood samples, and transvaginal ultrasound. Waist circumference was determined as the minimum circumference between the iliac crest and lower costae, whereas the hip circumference was determined as the maximum circumference over the gluteal region.

Sex hormones levels were determined as total testosterone, free testosterone and sex hormone-binding globulin (SHBG). The applied assays have previously been described [21,31]. In brief: Total testosterone was determined at Statens Serum Institute (Copenhagen, Denmark) by liquid chromatography tandem mass spectrometry. Free testosterone levels were calculated from total testosterone and SHBG levels according to Vermeulen et al. [32]. Free testosterone levels > 0.034 nmol/l were considered elevated levels. In Denmark, the normal range of free testosterone levels in fertile aged women is [0.006–0.034] nmol/l, which is used nationally. Insulin was analyzed by time-resolved fluoroimmunoassay with a commercial kit (AutoDELFIA; Wallac Oy, Turku, Finland). Plasma total cholesterol, high-density lipoprotein (HDL), cholesterol and triglyceride (TG) levels were analyzed by enzymatic colorimetric reactions (Modular P, Roche, Hvidovre, Denmark), and low-density lipoprotein (LDL) cholesterol was calculated using the Friedewald equation. Blood glucose was measured on capillary ear blood using Hemo Cue (Quest Diagnostics, Denmark).

### DNA purification and mutation analysis

Genomic DNA was isolated from peripheral leukocytes using a Maxwell®16 robot (Promega, Denmark). All exons and exon-intron boundaries of *DENND1A* (accession no. NM_020946.1) was analyzed by bi-directional sequencing using the Big Dye® Terminator v.3.1 cycle sequencing kit (Applied Biosystems, Denmark) and an ABI3730XL capillary sequencer (Applied Biosystems, Denmark). Specific forward and reverse primers were designed to align with exons and exon-intron boundaries of *DENND1A* (table S1).

### Single-nucleotide polymorphism assay

All patients and controls were tested for presence of the missense SNP rs189947178 (c.2351C>A p.Ala 784 Asp) using allelic discrimination by real time PCR (StepOnePlus^TM^ Real-time PCR Systems, Naerum, Denmark) and probe-based allelic discrimination assay (Pentabase, Odense, Denmark). The method takes advantage of the single base change C/A that discriminates between wild type and mutation. The real time PCR were carried out with probes designed specifically for either the wild type sequence (HEX-CCC TGC TCG CCC TCC-BHQ-1) or the mutation sequence (FAM-CCC TGC TCG ACC TCC T-BHQ-1), and specific primers flanking the region of interest (forward primer: 5´- TGC AGG CAC GAG CAG TGA - `3, reverse primer: 5´- AGG CTG TGC TGA GCG GGT - `3). Reactions were prepared in 96 wells plates as follows: 3.5 µl TaqMan Genotyping Master Mix (Applied Biosystems, Denmark), 1.26 µl forward primer (5 µM), 1.26 µl reverse primer (5 µM), 0.14 µl wild-type probe (5 µM), and 0.14 µl mutation probe (5 µM). The PCR conditions were: step 1: 10 min at 95 °C; step 2 (repeated 40 times): 5 s at 95 °C, 30 s at 60 °C; step 3: 30 s at 60 °C, step 4: 4 °C.

### Pathogenecity analysis of mutation

Database of Single Nucleotide Polymorphisms (dbSNP) (Bethesda, MD: National Center for Biotechnology Information, National Library of Medicine; dbSNP Build ID: 131, Genome Build 37.4) was used to retrieve information on minor allele frequencies (MAF) [33]. Pathogenecity analyses were performed with PolyPhen [34], Mutationtaster [35] and SIFT [36–40]. Polyphen, Mutationtaster and SIFT mainly do predictions based on the presence or absence of conservation of highly related sequences. The Alamut software (Alamut version 2.1, Interactive Biosoftware, Rouen, France) ESE and Splice site prediction tools were used for predicting protein function effects of nucleotide changes. The Research Collaboratory for Structural Bioinformatics PDB (http://www.pdb.org/pdb/home/home.do) was used to obtain protein crystal structures.

### Statistical analyses

The Mann-Whitney U test was used to compare clinical and subclinical characteristics and the data were presented as median (25–75 quartiles) using SPSS (SPSS version 19). Multiple regression analysis was applied to detect association of rs189947178 genotype distribution with PCOS status and with clinical variables, while controlling for age effects using the generalized linear model with a link function of binomial family. Model fitting was done using the *glm* function in the free R package *stats*. Adjustment for multiple comparisons was done using Bonferroni correction. After correction, the type I error rate was set at 0.004 (type I error at 0.05 divided by 13 sub-tests)*.*

## Results

Clinical and biochemical characteristics of sequenced patients with PCOS (n = 10) compared to unsequenced patients with PCOS (n = 155) are listed in table 1. Sequenced patients with PCOS was characterized by significantly higher weight, BMI, Ferriman-Gallwey score, free testosterone and triglyceride levels and lower SHBG levels compared to the additional patients with PCOS. Clinical and biochemical characteristics of the patients (n = 261) and controls are also listed in table 1. These results have partly been published before [16,21,22].

### Sequencing of *DENND1A*

**Figure 1:**
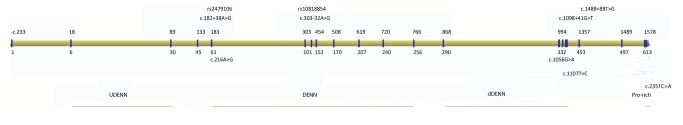
Distribution of the eight detected SNPs in *DENND1A*. The distribution of the eight SNPs detected by sequencing of *DENND1A* in patients with PCOS: rs1778890, rs9785285, rs12377595, rs116974312, rs61736953, rs3829851, rs10739633, rs189947178.

**Figure 2:**
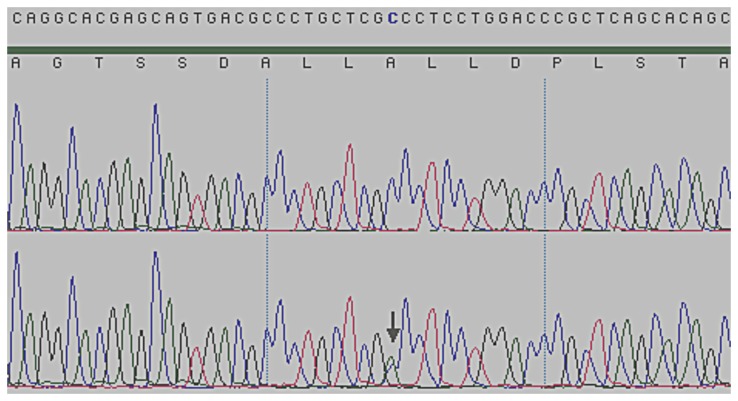
Chromatogram showing the rs189947178 variation (c.2351C>A, p.Ala784Asp) in exon 22 of *DENND1A.* Representation of the partial sequence of DENND1A exon 22 displaying in the upper pane a wild type sequence and in the lower pane the c.2351C>A allele (depicted by an arrow). Above the chromatograms the consensus protein and cDNA sequences are shown.

Eight SNPs were detected in the *DENND1A* exons and exon-intron boundary sequences (Figure 1). As depicted in Table 2, a number of genetic variants were observed in the sequenced PCOS patients. Seven of the eight SNPs detected were all synonymous or intron variants (Table 3). Further analysis using Alamut, predicted none of these seven synonymous/intron variants to influence DENND1A function. One patient harboured a missense SNP (c.2351C>A, p.Ala784Asp, rs189947178), replacing Alanine (Ala) with Aspartic acid (Asp). All of the eight SNPs detected are described in the dbSNP, hence we did not detect any new SNPs and thus the sequences of *DENND1A* were not uploaded. Figure 2 shows the chromatogram of the detected missense SNP, rs189947178. The MAF of patients with PCOS and moderate hirsutism (n = 27) was 0.056 (Table 3). According to dbSNP, the MAF of rs189947178 is 0.007.

**Table 2:** SNPs detected in *DENND1A* sequences of patients.

**cDNA position**	**Protein level**	**dbSNP**	**Location of SNP**	**dbSNP MAF**	**Patients MAF**
c.182+38A>G.	Intron	rs1778890	Intron	0.237	0.250
c.216A>G	p.Thr72Thr.	rs9785285	Exon 5	0.410	0.250
c.303-32A>G	Intron	rs12377595	Intron	0.271	0.250
c.1098+41C>A	Intron	rs116974312	Intron	0.033	0.100
c.1056G>A	p.Arg352Arg.	rs61736953	Exon 14	0.064	0.200
c.1107T>C	p.Asp369Asp	rs3829851	Exon 15	0.167	0.100
c.1488+88T>G	Intron	rs10739633	Intron	0.318	0.550
c.2351C>A	p.Ala784Asp	rs189947178	Exon 22	0.007	0.050

dbSNP: reference SNP number is indicated. Protein level: the result of nucleotide change on protein sequence is indicated when information was available. The allele frequency distributions were obtained from dbSNP (Genome Build 37.4).

**Table 3:** Genotype distribution and allele frequencies of rs189947178.

**Genotype**	**Controls (n = 248)**	**Patients (n = 261)**	**PCOS (n = 165)**	**Clinical hyperandrogenism (n = 96)**	**Patients with moderate hirsutism (n = 47)**	**PCOS with moderate hirsutism (n = 27)**
Carriers	2 (0.8)	5 (1.9)	4 (2.4)	1 (1.0)	3 (6.4)	3 (11.1)
Non-carriers	246 (99.2)	256 (98.1)	161 (97.5)	95 (99.0)	44 (93.6)	24 (88.9)
MAF	0.004	0.010	0.012	0.005	0.032	0.056
P-value	-	0.61	0.37	0.98	0.04	0.003*

Carriers: Subjects with the A allele (AC genotype).

Non-carriers: Subjects without the A allele (CC genotype).

PCOS was defined according to the Rotterdam criteria [2].

* P-values ≤ 0.004.

Pathogenecity analysis conducted with PolyPhen predicted the rs189947178 variant to be “Probably Damaging” with a score of 0.926. Mutationtaster and SIFT predicted the variant to be a polymorphism.

The classification made by Polyphen additionally includes information on tertiary structure and structural parameters. So far, no crystal structure of DENND1A has been published, but a crystal structure of the homologous DENND1B has been published as PDB: 3TW8 [41]. Despite the great homology, DENND1A and DENND1B varies in the C-terminal portion, thus an assessment of functional consequence could not be drawn from structural comparison. Garnier et al. [42] and Chou-Fasman’s [43] secondary structure evaluation predicted Ala784 to be part of an alpha helical amphipatic internal structure (data not shown). Thus, a change of the hydrophobic Ala784 for a polar amino acid might be damaging to protein function. Contrary to the highly conserved N-terminal domain DENN (uDENN), DENN and downstream DENN (dDENN) modules, the C-terminal tail shows large deviation between different DENN-containing molecules. In contrast to this, the C-terminal region of DENND1A shows strong phylogenetic conservation (data not shown), pointing to a distinct important function.

### Carrier status and allele frequencies of rs189947178

The AA genotype (homozygote mutation genotype) was not found in any patients or controls and table 3 shows carriers (AC genotype) and non-carriers (CC genotype) only.

Results for the all patients (n = 261) including the sequenced patients with PCOS (n = 10) and unsequenced patients with PCOS (n = 155) compared to controls (n = 248) are shown in table 3. There were no significant differences in the distribution of carriers versus non-carriers in: patients (n = 261) vs. controls (n = 248), patients with PCOS (n = 165) vs. controls (n = 248), patients with clinical hyperandrogenism (n = 96) or patients with moderate hirsutism (n = 47) vs. controls. There were significantly more carriers among patients with PCOS and moderate hirsutism (n = 27) compared to controls (P = 0.003). Multiple regression analysis was applied to evaluate carrier status of rs189947178. Ferriman-Gallwey score and PCOS status was not associated with carrier status (AC genotype, data not shown). In addition, no other clinical and biochemical parameters (testosterone levels, HDL, LDL, weight, fasting glucose, fasting insulin levels) were associated with carrier status of rs189947178 missense SNP.

The SNP rs2479106 previously found to be associated with PCOS and the missense SNP rs189947178 was not in linkage disequilibrium (D´= 0.201, r^2^ = 0).

## Discussion

In the present study, we sequenced the PCOS candidate gene, *DENND1A* to further investigate the association between PCOS and *DENND1A*, reported by us and others (11-15). Sequencing of the *DENND1A* gene from patients with PCOS identified eight SNPs within the coding region of *DENND1A*. One of these SNPs is a missense SNP (rs189947178) that gives rise to an amino acid change (alanine to aspartic acid) and therefore potentially could be associated with the PCOS pathogenesis.

The rs189947178 mutation variant leads to a change of a hydrophobic amino acid to a polar amino acid and therefore has a possibly damaging effect on the DENND1A protein. The involvement of DENND1A in endosomal membrane traffic could affect several organ systems as proposed by Goodarzi [14]. We therefore investigated if this mutation was only present in patients with PCOS and genotyped well characterized patients with PCOS or hirsutism and healthy controls. We found significantly more carriers among patients with PCOS and moderate hirsutism compared to controls. Due to the small sample size of patients with PCOS and moderate hirsutism, it is possible that the significant more carriers among patients with PCOS and moderate hirsutism was a false positive association – hence, the multiple regression analysis did not support an association between this SNP and Ferriman-Gallwey score or PCOS diagnosis.

A recent study of genotype-phenotype correlations of the GWAS-identified PCOS susceptibility SNP rs2479106, found an association between the GG + AG group of patients and elevated serum insulin after a glucose load [44]. The DENND1A protein is in part involved in endosomal recycling [19,20] and changes in the conformation could alter protein function, which hypothetically could affect insulin secretion. Our data did however not indicate any association of the rs189947178 missense carrier status and insulin levels.

Our findings regarding rs189947178 carrier status, along with previous findings of association between SNPs found in *DENND1A* [11,13–16], could indicate that these SNPs serves as genomic markers for alterations in genes separate from *DENND1A*. The SNP rs2479106 was associated with PCOS in several studies. rs2479106 is located in an intron of the *DENND1A* gene, thus not within a coding region. A study by Goodarzi found an association between rs10818854 located in *DENND1A*, but not rs2479106 (13). The association between PCOS and SNPs in *DENND1A* could instead relate to these SNPs being in linkage disequilibrium with variation in a co-localized gene encoding a microRNA (miR601), as suggested by Goodarzi (13). miR601 is involved in expression of actin cytoskeleton and down regulation of the Fas induced apoptosis pathway and expression of nuclear factor-kappaB transcription factor dependent reporter [45]. The fact that SNPs in *DENND1A* instead could relate to functional variation in other genes are a limitation to this study. However, since the association between SNPs in *DENND1A* has been replicated in several studies [12–16], we considered *DENND1A* a possible candidate gene for PCOS.

Further studies in investigating the prevalence of rs189947178 in additional, independent larger cohorts could be relevant to conduct. If these studies replicate our finding, conducting functional studies determining the precise molecular effect of the missense variation rs189947178 could be relevant. It is important to mention that only the coding regions of *DENND1A* were sequenced in this study. This is a limitation to the study since we may have missed functional non-coding regulatory variation within the gene.

In conclusion, sequences of the *DENND1A* gene from patients with PCOS did not reveal alterations that alone could be causing the PCOS pathogenesis. We found significantly more carriers of the missense SNP rs18994717 among patients with PCOS and moderate hirsutism. However, because of the small sample size and the lack of association between this SNP and Ferriman-Gallwey score or PCOS diagnosis, this may be a false positive association. The PCOS pathogenesis may be heterogenic and caused by low penetrant common variants in different genes. The rs189947178 missense SNP may represent a part of a rare PCOS genotype or it may be a marker for PCOS related genetic alterations located apart from *DENND1A*. Further studies are needed to clarify this.

## Supporting Information

Table S1 **Sequences of oligonucleotide forward and reverse primers for sequencing of the *DENND1A* gene.**
(DOC)

## References

[1] Azziz R, Woods KS, Reyna R, Key TJ, Knochenhauer ES et al. (2004) The prevalence and features of the polycystic ovary syndrome in an unselected population. J Clin Endocrinol Metab 89: 2745-2749. doi:10.1210/jc.2003-032046. PubMed: 15181052. 15181052 10.1210/jc.2003-032046

[2] The Rotterdam ESHRE/ASRM-Sponsored PCOS consensus workshop group (2004) Revised 2003 consensus on diagnostic criteria and long-term health risks related to polycystic ovary syndrome (PCOS). Hum Reprod 19: 41-47. doi:10.1093/humrep/deh098. PubMed: 14688154. doi:10.1093/humrep/deh098 PubMed: 14688154 14688154 10.1093/humrep/deh098

[3] Glintborg D, Henriksen JE, Andersen M, Hagen C, Hangaard J et al. (2004) Prevalence of endocrine diseases and abnormal glucose tolerance tests in 340 Caucasian premenopausal women with hirsutism as the referral diagnosis. Fertil Steril 82: 1570-1579. doi:10.1016/j.fertnstert.2004.06.040. PubMed: 15589862. 15589862 10.1016/j.fertnstert.2004.06.040

[4] Glintborg D, Andersen M (2010) An update on the pathogenesis, inflammation, and metabolism in hirsutism and polycystic ovary syndrome. Gynecol Endocrinol 26: 281-296. doi:10.3109/09513590903247873. PubMed: 20141388. 20141388 10.3109/09513590903247873

[5] Diamanti-Kandarakis E (2008) Polycystic ovarian syndrome: pathophysiology, molecular aspects and clinical implications. Expert Rev Mol Med 10: e3. doi:10.1017/S1462399408000598. PubMed: 18230193. 18230193 10.1017/S1462399408000598

[6] Eriksen M, Pørneki AD, Skov V, Burns JS, Beck-Nielsen H et al. (2010) Insulin resistance is not conserved in myotubes established from women with PCOS. PLOS ONE 5: e14469. doi:10.1371/journal.pone.0014469. PubMed: 21209881. 21209881 10.1371/journal.pone.0014469 PMC3012693

[7] Eriksen MB, Minet AD, Glintborg D, Gaster M (2011) Intact primary mitochondrial function in myotubes established from women with PCOS. J Clin Endocrinol Metab 96: E1298-E1302. doi:10.1210/jc.2011-0278. PubMed: 21593108. 21593108 10.1210/jc.2011-0278

[8] Ewens KG, Jones MR, Ankener W, Stewart DR, Urbanek M et al. (2011) Type 2 diabetes susceptibility single-nucleotide polymorphisms are not associated with polycystic ovary syndrome. Fertil Steril 95: 2538-2541. doi:10.1016/j.fertnstert.2011.02.050. PubMed: 21444075. 21444075 10.1016/j.fertnstert.2011.02.050 PMC3124609

[9] Jones MR, Chua AK, Mengesha EA, Taylor KD, Chen YD et al. (2012) Metabolic and cardiovascular genes in polycystic ovary syndrome: a candidate-wide association study (CWAS). Steroids 77: 317-322. doi:10.1016/j.steroids.2011.12.005. PubMed: 22178785. 22178785 10.1016/j.steroids.2011.12.005 PMC3689580

[10] Ewens KG, Jones MR, Ankener W, Stewart DR, Urbanek M et al. (2011) FTO and MC4R gene variants are associated with obesity in polycystic ovary syndrome. PLOS ONE 6: e16390. doi:10.1371/journal.pone.0016390. PubMed: 21283731. 21283731 10.1371/journal.pone.0016390 PMC3024473

[11] Chen ZJ, Zhao H, He L, Shi Y, Qin Y et al. (2011) Genome-wide association study identifies susceptibility loci for polycystic ovary syndrome on chromosome 2p16.3, 2p21 and 9q33.3. Nat Genet 43: 55-59. doi:10.1038/ng.732. PubMed: 21151128. 21151128 10.1038/ng.732

[12] Shi Y, Zhao H, Shi Y, Cao Y, Yang D et al. (2012) Genome-wide association study identifies eight new risk loci for polycystic ovary syndrome. Nat Genet 44: 1020-1025. doi:10.1038/ng.2384. PubMed: 22885925. 22885925 10.1038/ng.2384

[13] Lerchbaum E, Trummer O, Giuliani A, Gruber HJ, Pieber TR et al. (2011) Susceptibility Loci for polycystic ovary syndrome on chromosome 2p16.3, 2p21, and 9q33.3 in a cohort of caucasian women. Horm Metab Res 43: 743-747. doi:10.1055/s-0031-1286279. PubMed: 22009367. 22009367 10.1055/s-0031-1286279

[14] Goodarzi MO, Jones MR, Li X, Chua AK, Garcia OA et al. (2012) Replication of association of DENND1A and THADA variants with polycystic ovary syndrome in European cohorts. J Med Genet 49: 90-95. doi:10.1136/jmedgenet-2011-100427. PubMed: 22180642. 22180642 10.1136/jmedgenet-2011-100427 PMC3536488

[15] Welt CK, Styrkarsdottir U, Ehrmann DA, Thorleifsson G, Arason G et al. (2012) Variants in DENND1A Are Associated with Polycystic Ovary Syndrome in Women of European Ancestry. J Clin Endocrinol Metab 97: E1342-E1347. doi:10.1210/jc.2011-3478. PubMed: 22547425. 22547425 10.1210/jc.2011-3478 PMC3387396

[16] Eriksen MB, Brusgaard K, Andersen M, Tan Q, Altinok ML et al. (2012) Association of polycystic ovary syndrome susceptibility single nucleotide polymorphism rs2479106 and PCOS in Caucasian patients with PCOS or hirsutism as referral diagnosis. Eur J Obstet Gynecol Reprod Biol 163: 39-42. doi:10.1016/j.ejogrb.2012.03.020. PubMed: 22504079. 22504079 10.1016/j.ejogrb.2012.03.020

[17] Marat AL, Dokainish H, McPherson PS (2011) DENN domain proteins: regulators of Rab GTPases. J Biol Chem 286: 13791-13800. doi:10.1074/jbc.R110.217067. PubMed: 21330364. 21330364 10.1074/jbc.R110.217067 PMC3077579

[18] Allaire PD, Ritter B, Thomas S, Burman JL, Denisov AY et al. (2006) Connecdenn, a novel DENN domain-containing protein of neuronal clathrin-coated vesicles functioning in synaptic vesicle endocytosis. J Neurosci 26: 13202-13212. doi:10.1523/JNEUROSCI.4608-06.2006. PubMed: 17182770. 17182770 10.1523/JNEUROSCI.4608-06.2006 PMC6674997

[19] Allaire PD, Marat AL, Dall’Armi C, Di PG, McPherson PS et al. (2010) The Connecdenn DENN domain: a GEF for Rab35 mediating cargo-specific exit from early endosomes. Mol Cell 37: 370-382. doi:10.1016/j.molcel.2009.12.037. PubMed: 20159556. 20159556 10.1016/j.molcel.2009.12.037 PMC2825348

[20] Kouranti I, Sachse M, Arouche N, Goud B, Echard A (2006) Rab35 regulates an endocytic recycling pathway essential for the terminal steps of cytokinesis. Curr Biol 16: 1719-1725. doi:10.1016/j.cub.2006.07.020. PubMed: 16950109. 16950109 10.1016/j.cub.2006.07.020

[21] Glintborg D, Mumm H, Hougaard DM, Ravn P, Andersen M (2012) Smoking is associated with increased adrenal responsiveness, decreased prolactin levels and a more adverse lipid profile in 650 white patients with polycystic ovary syndrome. Gynecol Endocrinol 28: 170-174. doi:10.3109/09513590.2011.589926. PubMed: 21770838. 21770838 10.3109/09513590.2011.589926

[22] Glintborg D, Hermann AP, Brusgaard K, Hangaard J, Hagen C et al. (2005) Significantly higher adrenocorticotropin-stimulated cortisol and 17-hydroxyprogesterone levels in 337 consecutive, premenopausal, caucasian, hirsute patients compared with healthy controls. J Clin Endocrinol Metab 90: 1347-1353. PubMed: 15598692. 15598692 10.1210/jc.2004-1214

[23] Téllez R, Frenkel J (1995) [Clinical evaluation of body hair in healthy women]. Rev Med Chil 123: 1349-1354. PubMed: 8733277. 8733277

[24] Asunción M, Calvo RM, San Millán JL, Sancho J, Avila S et al. (2000) A prospective study of the prevalence of the polycystic ovary syndrome in unselected Caucasian women from Spain. J Clin Endocrinol Metab 85: 2434-2438. doi:10.1210/jc.85.7.2434. PubMed: 10902790. 10902790 10.1210/jcem.85.7.6682

[25] Sagsoz N, Kamaci M, Orbak Z (2004) Body hair scores and total hair diameters in healthy women in the Kirikkale Region of Turkey. Yonsei Med J 45: 483-491. PubMed: 15227736. 15227736 10.3349/ymj.2004.45.3.483

[26] DeUgarte CM, Woods KS, Bartolucci AA, Azziz R (2006) Degree of facial and body terminal hair growth in unselected black and white women: toward a populational definition of hirsutism. J Clin Endocrinol Metab 91: 1345-1350. doi:10.1210/jc.2004-2301. PubMed: 16449347. 16449347 10.1210/jc.2004-2301

[27] Api M, Badoglu B, Akca A, Api O, Gorgen H et al. (2009) Interobserver variability of modified Ferriman-Gallwey hirsutism score in a Turkish population. Arch Gynecol Obstet 279: 473-479. doi:10.1007/s00404-008-0747-8. PubMed: 18677501. 18677501 10.1007/s00404-008-0747-8

[28] Moran C, Tena G, Moran S, Ruiz P, Reyna R et al. (2010) Prevalence of polycystic ovary syndrome and related disorders in mexican women. Gynecol Obstet Invest 69: 274-280. doi:10.1159/000277640. PubMed: 20110726. 20110726 10.1159/000277640

[29] Escobar-Morreale HF, Carmina E, Dewailly D, Gambineri A, Kelestimur F et al. (2012) Epidemiology, diagnosis and management of hirsutism: a consensus statement by the Androgen Excess and Polycystic Ovary Syndrome Society. Hum Reprod Update 18: 146-170. doi:10.1093/humupd/dmr042. PubMed: 22064667. 22064667 10.1093/humupd/dmr042

[30] Cook H, Brennan K, Azziz R (2011) Reanalyzing the modified Ferriman-Gallwey score: is there a simpler method for assessing the extent of hirsutism? Fertil Steril 96: 1266-1270. doi:10.1016/j.fertnstert.2011.08.022. PubMed: 21924716. 21924716 10.1016/j.fertnstert.2011.08.022 PMC3205229

[31] Glintborg D, Mumm H, Hougaard D, Ravn P, Andersen M (2010) Ethnic differences in Rotterdam criteria and metabolic risk factors in a multiethnic group of women with PCOS studied in Denmark. Clin Endocrinol (Oxf) 73: 732-738. doi:10.1111/j.1365-2265.2010.03873.x. 20846294 10.1111/j.1365-2265.2010.03873.x

[32] Vermeulen A, Verdonck L, Kaufman JM (1999) A critical evaluation of simple methods for the estimation of free testosterone in serum. J Clin Endocrinol Metab 84: 3666-3672. doi:10.1210/jc.84.10.3666. PubMed: 10523012. 10523012 10.1210/jcem.84.10.6079

[33] Sherry ST, Ward MH, Kholodov M, Baker J, Phan L et al. (2001) dbSNP: the NCBI database of genetic variation. Nucleic Acids Res 29: 308-311. doi:10.1093/nar/29.1.308. PubMed: 11125122. 11125122 10.1093/nar/29.1.308 PMC29783

[34] Adzhubei IA, Schmidt S, Peshkin L, Ramensky VE, Gerasimova A et al. (2010) A method and server for predicting damaging missense mutations. Nat Methods 7: 248-249. doi:10.1038/nmeth0410-248. PubMed: 20354512. 20354512 10.1038/nmeth0410-248 PMC2855889

[35] Schwarz JM, Rödelsperger C, Schuelke M, Seelow D (2010) MutationTaster evaluates disease-causing potential of sequence alterations. Nat Methods 7: 575-576. doi:10.1038/nmeth0810-575. PubMed: 20676075. 20676075 10.1038/nmeth0810-575

[36] Ng PC, Henikoff S (2001) Predicting deleterious amino acid substitutions. Genome Res 11: 863-874. doi:10.1101/gr.176601. PubMed: 11337480. 11337480 10.1101/gr.176601 PMC311071

[37] Ng PC, Henikoff S (2002) Accounting for human polymorphisms predicted to affect protein function. Genome Res 12: 436-446. doi:10.1101/gr.212802. PubMed: 11875032. 11875032 10.1101/gr.212802 PMC155281

[38] Ng PC, Henikoff S (2003) SIFT: Predicting amino acid changes that affect protein function. Nucleic Acids Res 31: 3812-3814. doi:10.1093/nar/gkg509. PubMed: 12824425. 12824425 10.1093/nar/gkg509 PMC168916

[39] Ng PC, Henikoff S (2006) Predicting the effects of amino acid substitutions on protein function. Annu Rev Genomics Hum Genet 7: 61-80. doi:10.1146/annurev.genom.7.080505.115630. PubMed: 16824020. 16824020 10.1146/annurev.genom.7.080505.115630

[40] Kumar P, Henikoff S, Ng PC (2009) Predicting the effects of coding non-synonymous variants on protein function using the SIFT algorithm. Nat Protoc 4: 1073-1081. doi:10.1038/nprot.2009.86. PubMed: 19561590. 19561590 10.1038/nprot.2009.86

[41] Wu X, Bradley MJ, Cai Y, Kümmel D, De La Cruz EM et al. (2011) Insights regarding guanine nucleotide exchange from the structure of a DENN-domain protein complexed with its Rab GTPase substrate. Proc Natl Acad Sci U S A 108: 18672-18677. doi:10.1073/pnas.1110415108. PubMed: 22065758. 22065758 10.1073/pnas.1110415108 PMC3219131

[42] Garnier J, Osguthorpe DJ, Robson B (1978) Analysis of the accuracy and implications of simple methods for predicting the secondary structure of globular proteins. J Mol Biol 120: 97-120. doi:10.1016/0022-2836(78)90297-8. PubMed: 642007. 642007 10.1016/0022-2836(78)90297-8

[43] Chou PY, Fasman GD (1974) Prediction of protein conformation. Biochemistry 13: 222-245. doi:10.1021/bi00699a002. PubMed: 4358940. 4358940 10.1021/bi00699a002

[44] Cui L, Zhao H, Zhang B, Qu Z, Liu J et al. (2013) Genotype-phenotype correlations of PCOS susceptibility SNPs identified by GWAS in a large cohort of Han Chinese women. Hum Reprod 28: 538-544. doi:10.1093/humrep/des424. PubMed: 23208300. 23208300 10.1093/humrep/des424

[45] Ohdaira H, Nakagawa H, Yoshida K (2009) Profiling of molecular pathways regulated by microRNA 601. Comput Biol Chem 33: 429-433. doi:10.1016/j.compbiolchem.2009.09.003. PubMed: 19889580. 19889580 10.1016/j.compbiolchem.2009.09.003

