# Supplementary material for: Genetic Alterations within the DENND1A Gene in Patients with Polycystic Ovary Syndrome (PCOS)
Source: PLoS One. 2013 Sep 27;8(9):e77186. doi: 10.1371/journal.pone.0077186 (PMC3785455; doi:10.1371/journal.pone.0077186)
Supplement: Table S1 — Sequences of oligonucleotide forward and reverse primers for sequencing of the DENND1A gene. (DOC) [file pone.0077186.s001.doc]

| **Exon** | **Forward primer** | **Reverse primer** |
| --- | --- | --- |
| **1** | ggcgttcgccgggctgga | cccctctggtctcggcaacaagtc |
| **2** | tctataccatgctgcctcaatctt | cacgtgcatagctatctcaaacaa |
| **3** | gagtgccttgccgagtcctt | aacaccccaaatctagcaccaata |
| **4** | agcccatatttcttcatctgt | tgccatacaaaatacatctcac |
| **5** | agcgcgagggttaaaatccag | gaagtccttctccccaaatgca |
| **6** | attagcatttcgccagtttgtgt | tatttattttagggaagggagtgg |
| **7** | ataaaaggcggtcatggtaggtgt | taggggaggtatggctgacaaaat |
| **8** | gccttggcctcccgaaatg | caaacaaataggcagaaaacagagaaga |
| **9** | tgcgacctcatgttgtaatcacc | tgctgccatataaggggactgtag |
| **10** | tgctgaaatcaaatggcggtgtc | atcagggcaaaggagaagcagagg |
| **11** | tgcccttcatccaacctta | agtgcacggcgtccaaacc |
| **12** | acacagccgtatatcagcattcct | ttccccattccccaaagtcac |
| **13** | aatggcgcaatctcggctcac | acgcggttggggcttactgtcta |
| **14a** | gctacccgggaggctgagg | caaagaaaagggggaaaaaccatt |
| **14b** | agccgatcactttctgtgagg | tagagtggagacagctgcagaa |
| **14c** | gccttcgtgtcccactacc | tagagtggagacagctgcagaaa |
| **15** | gggagggggattggatgaaga | gccccaataaaggaaaagcaagtc |
| **16** | gtgaaaccccgtctctagtaaaaat | ccccccgaatgctctaaga |
| **17** | ggcagggaccagttatcat | gagcccaggagttcaagac |
| **18** | ggccctgccctggtgaaatagaat | gtggggtgggcaagggacag |
| **19** | ctggcaggcctcttcgttgtg | gcccgggatgctaaagtgttctaa |
| **20** | cctgcaaggaggccaatggaactat | agccccggggaagcctcaca |
| **21a** | ctgaaggcctgggtgggagagtgc | ccgagcggggatgtgatggattag |
| **21b** | ggattatgattgcccccttatgga | gacaccggctctttggcacac |
| **22a** | gcgggtgcctgcgatctaaaaagt | gggggtgaatggggtggctaca |
| **22b** | gccccgccaccccgaatgtag | tcgggctcacgtcttgcttggttt |
| **22c** | ccccgccaccccgaatgtagc | cctcggaaccgcagcagtggac |
